# Multidisciplinary collaborative treatment of a rare case of chronic hemoptysis: diagnostic and therapeutic insights from a 7-year clinical course

**DOI:** 10.3389/fmed.2026.1785345

**Published:** 2026-07-07

**Authors:** Jianxiong Kang, Peiyan Hua

**Affiliations:** 1 People's Hospital of Chuxiong Yi Autonomous Prefecture, Chuxiong, China; 2 Department of Thoracic, The Second Hospital of Jilin University, Changchun, Jilin, China

**Keywords:** angiogenesis, Aspergillus, hemoptysis, pulmonary artery ligation, tuberculosis

## Abstract

A middle-aged woman who had undergone a right upper and middle lobectomy 7 years earlier presented with recurrent intermittent hemoptysis. Evaluation at our institution revealed that the main right pulmonary artery supplying the remaining lung had previously been transected and ligated. She also had pulmonary aspergillosis, tuberculosis, and bronchiectasis. Sequential interventions, including bronchoscopy and bronchial artery embolization (BAE) with gelatin sponge particles (350–560 μm), failed to achieve durable hemostasis. Double-lumen endotracheal intubation was performed to prevent blood spillover into the contralateral lung and avoid asphyxia. Definitive surgical treatment was therefore performed. To the best of our knowledge, this is the first reported case to combine iatrogenic main pulmonary artery transection during lobectomy, 7-year survival of the residual lung sustained by multi-source systemic collateral perfusion, coexisting pulmonary aspergillosis and tuberculosis, and COVID-19-triggered life-threatening hemoptysis. This case illustrates a complex, multifactorial mechanism underlying massive hemoptysis. Following right pulmonary artery interruption, the residual lung became dependent on systemic collateral circulation, including hypertrophied bronchial arteries and non-bronchial systemic arteries (NBSAs). These high-pressure, fragile vessels—together with chronic infection and structural lung disease, particularly Aspergillus colonization and post-tuberculous changes—promote vascular remodeling, erosion, and rupture. Superimposed viral infection (e.g., COVID-19) may further exacerbate inflammation and vascular instability, triggering life-threatening bleeding. Given its rarity and complexity, this case provides important insights into the mechanisms and management challenges of refractory hemoptysis driven by multi-source systemic collaterals.

## Introduction

**Figure 1:**
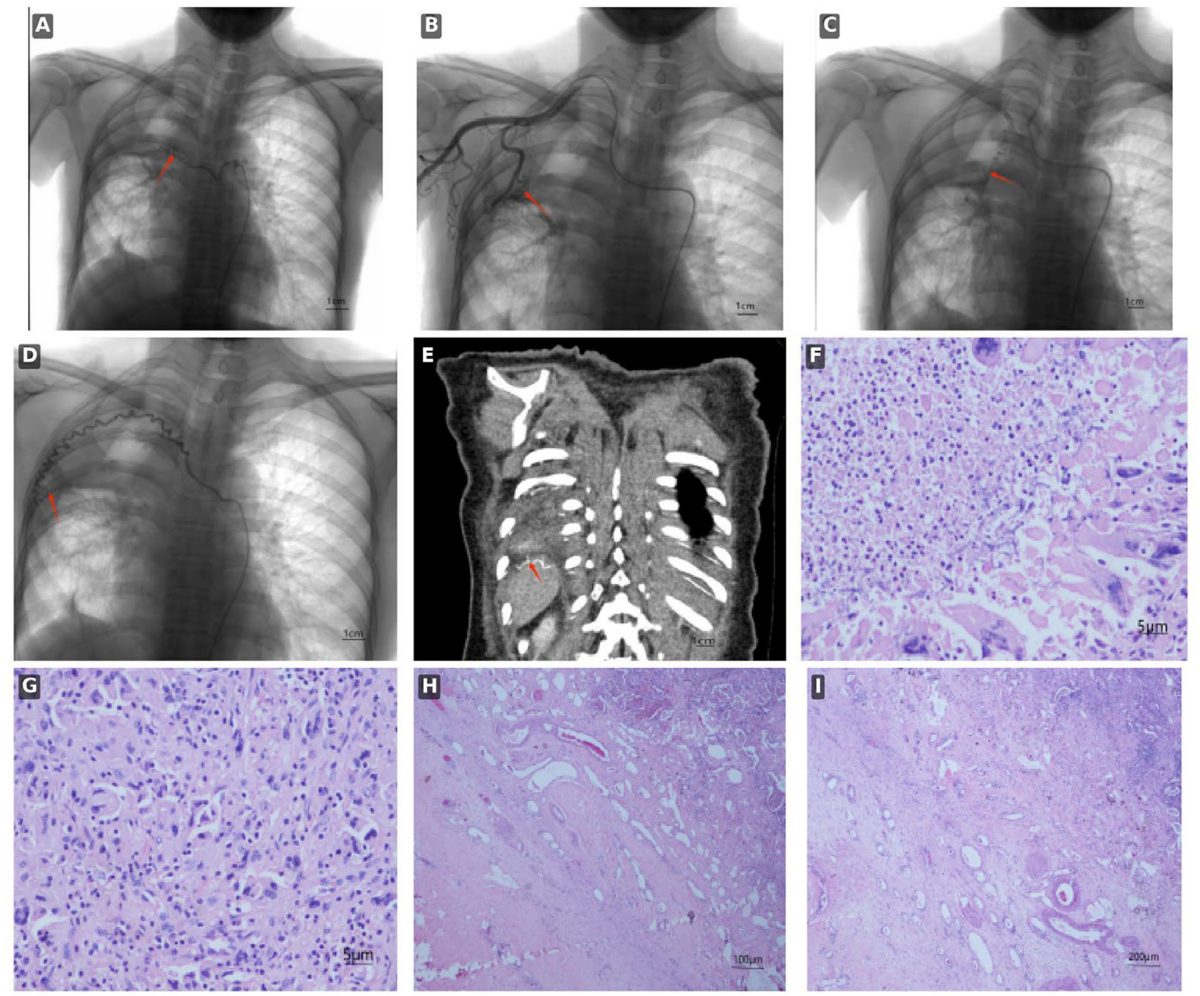
Radiographic and pathological findings of the patient. **(A)** Digital subtraction angiography (DSA) demonstrating a dilated and tortuous bronchial artery (red arrow) supplying the right lower lobe. **(B)** DSA showing collateral supply from the right subclavian artery (red arrow) to the right lower lobe. **(C)** DSA demonstrating collateral supply from the right internal thoracic artery (red arrow). **(D)** DSA showing collateral supply from the intercostal artery (red arrow). **(E)** Coronal contrast-enhanced computed tomography (CT) demonstrating collateral supply from the right inferior phrenic artery (red arrow) to the residual right lower lobe. **(F)** Hematoxylin and eosin (H&E) staining of the resected lung tissue showing Aspergillus hyphae (arrow; scale bar = 5 μm). **(G)** H&E staining demonstrating interstitial fibrous tissue proliferation, extensive inflammatory cell infiltration, and the presence of multinucleated giant cells (scale bar = 5 μm). **(H)** H&E staining showing pleural fibrous tissue hyperplasia (scale bar = 100 μm). **(I)** H&E staining demonstrating pleural thickening with fibrous tissue proliferation, fibrosis, and hyaline degeneration (scale bar = 200 μm).

Accidental ligation of the pulmonary artery during surgery is rare. Interruption of the main pulmonary arterial supply is expected to cause ischemia, thereby limiting the viability of the residual lung. We report a case of pulmonary artery ligation complicated by pulmonary aspergillosis, tuberculosis, and recurrent hemoptysis. Notably, the residual lung was maintained by extensive systemic collateralization, including hypertrophied bronchial arteries and multiple non-bronchial systemic arteries (NBSAs). Previous studies have shown that, in patients with hemoptysis related to bronchiectasis and chronic infection who undergo bronchial artery embolization, up to 66% have NBSAs as culprit vessels (1). Tuberculosis accounts for approximately 10% of hemoptysis cases (2), and hemoptysis occurs in 63.8% of chronic pulmonary aspergillosis cases (3). The novelty of this case lies in the combination of four features: 1. iatrogenic transection of the main pulmonary artery during lobectomy with prolonged (7-year) survival of the residual lung; 2. development of multi-source systemic collateral perfusion involving at least five arterial territories; 3. coexisting pulmonary aspergillosis and tuberculosis as compounding factors; and 4. SARS-CoV-2 infection triggering life-threatening hemoptysis. In this setting, the residual lung becomes dependent on systemic collateral perfusion after pulmonary artery transection, leading to fragile, high-pressure vascular networks. These vessels are prone to rupture, particularly in the presence of chronic inflammation associated with tuberculosis and structural lung disease. Aspergillus colonization further promotes vascular invasion and erosion, while superimposed viral infection may exacerbate airway inflammation and vascular instability, collectively precipitating massive hemoptysis. This report highlights the combined roles of bronchial arteries, NBSAs, tuberculosis, and aspergillosis in this rare scenario, with the aim of improving the understanding and management of refractory, life-threatening hemoptysis (Figure 1).

## Case report

We report the case of a 49-year-old female patient who was admitted to our hospital for further treatment due to aggravated hemoptysis. She had received 1 week of symptomatic treatment for intermittent hemoptysis in a primary hospital before transfer, and her general condition was poor, presenting with cachexia and anemia. Seven years prior to admission, the patient underwent right upper and middle lobectomy due to a right upper lung mass. During the operation, the right pulmonary artery trunk was accidentally transected. Postoperatively, the patient required 45 days of intensive care unit (ICU) treatment due to a critical condition, and intermittent hemoptysis occurred during this period. Since discharge, she has had recurrent intermittent hemoptysis, which was mostly induced by respiratory tract infections. Throughout the 7-year interval, the patient received no antifungal therapy. The current hemoptysis attack was secondary to SARS-CoV-2 infection, with significantly more severe symptoms than previous episodes. Chest computed tomography (CT) showed right pneumonia, bronchiectasis, and old tuberculous lesions in both lungs, suggesting that the bleeding source was located in the right lung. After admission, initial treatments included anti-infection, hemostasis, sedation, and mechanical ventilation. Double-lumen endotracheal intubation was performed to prevent blood spillover from the hemorrhagic right lung into the contralateral healthy left lung and subsequent asphyxia, and sputum culture was performed to identify the causative pathogens. After 2 days of conservative treatment, the patient’s hemoptysis was not controlled, with 2–3 episodes per day and a single bleeding volume of 100–300 mL, which was life-threatening. Therefore, endovascular interventional therapy was performed to locate the bleeding site and implement embolization hemostasis. Bronchial arteriography confirmed that the bleeding originated from the dilated and tortuous bronchial artery. Combined with imaging and anatomical analysis, the patient was diagnosed with iatrogenic right pulmonary artery interruption (secondary to intraoperative transection), and the right lower lobe was perfused by collateral vessels arising from the bronchial artery, right subclavian artery, internal thoracic artery, intercostal artery, and right inferior phrenic artery. The bleeding bronchial artery was selectively catheterized and embolized using gelatin sponge particles (350–560 μm) as the embolic material, achieving angiographic occlusion of the target vessel; however, hemoptysis was only temporarily relieved after the procedure. Subsequent sputum culture and etiological examination confirmed a pulmonary Aspergillus infection. Antifungal therapy with intravenous amphotericin B was initiated and continued for 1 week; however, despite temporary improvement after interventional therapy, the patient quickly developed recurrent massive hemoptysis, and conservative and interventional treatments failed to achieve sustained hemostasis, placing the patient at high risk of death. Multidisciplinary consultation (thoracic surgery, anesthesiology, and intensive care medicine) determined that the non-functional right lower lobe was the source of refractory bleeding, and surgical resection was the only radical treatment. Preoperative contrast-enhanced chest CT showed right thoracic cavity collapse, intercostal space narrowing, severe pleural thickening and adhesion, and multiple systemic collateral arteries supplying the residual right lung tissue. The main surgical risks included intraoperative massive bleeding caused by severe adhesion and anatomical variation and asphyxia induced by hemoptysis during the operation. The anesthetic strategy was primarily designed to avert asphyxia caused by intraoperative massive hemoptysis. Before surgery, fiberoptic bronchoscopy was carried out to clear airway blood clots. Under direct visualization, a double-lumen endotracheal tube and a bronchial blocker were inserted to achieve complete isolation of the hemorrhagic lung. Meanwhile, rapid sequence induction was applied to prevent cough and pulmonary aspiration during the anesthesia induction phase. The operation was performed via a 15-cm incision in the fifth intercostal space in the midaxillary line. Severe dense adhesion between the lung and chest wall was found intraoperatively, resulting in massive bleeding during dissection, which was treated with autologous blood transfusion. Due to thoracic cavity collapse and hilar tissue fusion, extrapericardial vascular dissection could not be completed; therefore, pericardiotomy was performed to transect the residual pulmonary artery and inferior pulmonary vein within the pericardial cavity, and the residual right lower lobe was successfully resected. After the operation, the patient was transferred to the ICU for close monitoring. No recurrent hemoptysis was observed postoperatively. On postoperative day 2, the tracheal intubation was removed, and mechanical ventilation was weaned as respiratory function recovered; on postoperative day 3, the chest drainage tube was removed; and on postoperative day 4, the patient was transferred to the general ward. Pathological examination of the resected lung tissue confirmed Aspergillus infection, with inflammatory vascular malformation and neovascularization in the lesion, which was consistent with the cause of recurrent hemoptysis. The patient recovered smoothly and was discharged 10 days after the operation. No recurrence of hemoptysis was observed during the 6-month follow-up.

## Discussion

Inadvertent transection of the main pulmonary artery during pulmonary resection is a catastrophic intraoperative complication in thoracic surgery, often requiring immediate vascular repair or completion pneumonectomy to prevent fatal ischemia (4, 5). Long-term survival of ipsilateral lung parenchyma without repair is exceedingly rare. To the best of our knowledge, no prior report has described the combination of iatrogenic main pulmonary artery transection during lobectomy, prolonged (7-year) survival of the residual lung via multi-source systemic collateral perfusion, coexisting pulmonary aspergillosis and tuberculosis, and COVID-19-triggered life-threatening hemoptysis. This constellation of findings defines the novelty of the present case and distinguishes it from previously reported cases of pulmonary artery ligation. In this case, preservation of the right lower lobe can be explained by the lung’s dual blood supply and subsequent adaptive hemodynamic changes. Under physiological conditions, the pulmonary artery provides approximately 95% of pulmonary perfusion, whereas the bronchial arteries—systemic vessels operating at pressures of approximately 90 mmHg—contribute only 1–2% of cardiac output and primarily serve a nutritive role (6). After acute pulmonary artery occlusion, bronchial arterial flow increases rapidly. Experimental studies have shown that flow increases within 4 days, the vessel diameter expands to approximately three times baseline within 1 week, and flow reaches its peak at 4 weeks. In canine models, hypertrophied bronchial arteries can supply up to two-thirds of normal pulmonary arterial flow (7). Additionally, in the acute phase, retrograde perfusion from the left atrium via the pulmonary veins may provide transient supplementary blood flow. Reduced pulmonary venous pressure creates a gradient that allows reverse flow sufficient to meet the immediate metabolic demands of ischemic lung tissue (8). Comparable reports include a patient with Behçet’s disease who survived long-term after pulmonary artery ligation and another who survived 35 years after inadvertent ligation of the left pulmonary artery during ductal ligation surgery (9, 10). However, neither involved iatrogenic transection during lobectomy, underscoring the rarity of this case. The progressive vascular remodeling that sustained the right lower lobe over 7 years ultimately created the anatomical basis for the development of refractory hemoptysis.

The second key issue is how the right lower lobe became perfused by a complex network of non-bronchial systemic arteries (NBSAs), including branches from the right subclavian, internal thoracic, intercostal, and right inferior phrenic arteries, as demonstrated by bronchial arteriography and contrast-enhanced CT. Under normal conditions, NBSAs do not supply the pulmonary parenchyma (11). The development of this collateral circulation can be understood as a two-stage process. First, transection of the main pulmonary artery causes an abrupt loss of distal pulmonary arterial pressure, creating a marked gradient between the systemic and pulmonary circulations. This results in compensatory hypertrophy and recruitment of bronchial arteries, as described in chronic pulmonary artery obstruction such as chronic thromboembolic pulmonary hypertension (CTEPH) (6, 12). As systemic inflow progressively replaces pulmonary perfusion, the affected lung becomes dependent on high-pressure collateral circulation. Second, a chronic inflammatory microenvironment—due to coexisting pulmonary tuberculosis and Aspergillus infection—combined with pleural thickening and adhesions after prior right upper and middle lobectomy, promotes NBSA recruitment. Pleural thickening and adhesions facilitate ingrowth of parietal pleural vessels (from intercostal, internal thoracic, and subclavian arteries) into the visceral pleura and lung parenchyma (13, 14). On contrast-enhanced CT, pleural thickness >3 mm with enhancing vessels in the extrapleural fat layer is a recognized indicator of NBSA supply (13, 14). However, pleural adhesion is not essential; experimental studies have shown that intercostal arteries can invade ischemic lung even after pulmonary artery ligation via sternotomy (15, 16). This process is believed to involve upregulation of vascular endothelial growth factor (VEGF) and pro-inflammatory cytokines, which promote endothelial migration and neovascularization (15, 16, 32). These mechanisms were not directly measured in this patient and should be considered as inferential findings. Histopathology of the resected specimen demonstrated inflammatory vascular malformation and extensive neovascularization, supporting this multi-stage angiogenic process. However, these collateral vessels lack the structural integrity of native pulmonary vasculature and are exposed to systemic pressures, making them fragile and rupture-prone. In the setting of chronic infection and recurrent inflammation—particularly Aspergillus-related angioinvasion and superimposed SARS-CoV-2 infection—this unstable network is highly susceptible to disruption. This explains the patient’s recurrent hemoptysis and eventual progression to life-threatening bleeding.

The third critical aspect of this case is the role of pulmonary aspergillosis as both a driver of vascular remodeling and the immediate trigger of life-threatening hemoptysis. The patient’s pre-existing post-tuberculous lesions provided a well-recognized substrate for Aspergillus colonization, as residual cavities offer an optimal environment for fungal growth (17). The preceding SARS-CoV-2 infection likely further impaired local host defenses, facilitating fungal proliferation, consistent with the known association between COVID-19 and pulmonary aspergillosis. Hemoptysis is the most common and potentially fatal manifestation of pulmonary aspergilloma, occurring in 50–90% of cases, with massive hemoptysis responsible for 2–10% of related mortality (18, 19). The main mechanisms of bleeding include mechanical irritation of the fungal ball against the vascular cavity wall, release of proteolytic enzymes causing vascular necrosis, and inflammatory angiogenesis that produces fragile neovessels within and around the cavity (2, 31). In conventional aspergilloma, the bronchial artery is the predominant source of bleeding (20). In contrast, this case involves Aspergillus infection superimposed on an extensive, multi-source systemic collateral circulation that developed after pulmonary artery transection. The Aspergillus-induced inflammatory response further weakened these pre-existing collateral vessels, which were already thin-walled, tortuous, and structurally fragile. This “double-hit” effect—chronic collateral remodeling combined with fungal-mediated vascular injury—likely explains the markedly increased severity of hemoptysis after SARS-CoV-2 infection and fungal activation, as well as the unusually large bleeding volume compared with that seen in isolated aspergilloma. Postoperative pathology demonstrating inflammatory vascular malformation and neovascularization provides direct support for this combined mechanism.

The fourth key issue concerns therapeutic decision-making, particularly the roles of bronchial artery embolization (BAE) and surgical resection. BAE is a well-established first-line treatment for massive hemoptysis, with immediate technical success rates of 85–98% (21, 22). When bleeding arises from the bronchial circulation, it is a safe, minimally invasive option and should generally precede surgery (21, 23). However, recurrence rates are higher in aspergilloma-associated hemoptysis, reaching 50–60% in some series (24, 25). This reflects the underlying disease rather than a limitation of the technique. BAE controls culprit vessels but does not eliminate the aspergilloma, which continues to cause mechanical irritation and enzymatic vascular damage. Persistent infection also promotes angiogenesis and new collateral formation, while multiple NBSA feeders make complete embolization difficult (1, 24, 26). In this case, BAE failure was due to disease complexity—specifically, at least five independent systemic arterial supplies to the right lower lobe—rather than technical inadequacy. The presence of NBSAs is an independent predictor of BAE failure and recurrence (1, 27, 28) and should not be generalized to simpler cases. Based on this case and the literature, a practical framework can be proposed. BAE is appropriate as first-line therapy when: 1. the bleeding source arises from bronchial or accessible systemic arteries; 2. the patient is hemodynamically stable; and 3. the underlying condition is amenable to embolization (e.g., bronchiectasis or limited aspergilloma without extensive collateralization). Surgery should be considered when: 1. BAE fails or hemoptysis recurs early; 2. the bleeding source is localized and resectable; 3. multi-source NBSA supply precludes complete embolization; and 4. bleeding is immediately life-threatening with no alternative options. In our patient, surgical indications were clear: failed BAE with early recurrence of life-threatening hemoptysis, a well-defined and resectable source (the non-functional right lower lobe), and extensive multi-source collateralization. Surgery in this setting carries substantial risk and should be reserved for selected patients. Operative challenges—including dense pleural adhesions, thoracic cavity contraction, and extensive collateral vessels—were addressed through careful planning, intraoperative autologous transfusion, and pericardiotomy to obtain intrapericardial control of the pulmonary artery stump and inferior pulmonary vein when extrapericardial dissection was not feasible. Anesthetic management was equally critical. Preoperative fiberoptic bronchoscopy was used to clear airway clots. Double-lumen intubation with bronchial blocker placement under direct visualization allowed lung isolation, and rapid-sequence induction minimized aspiration risk. This coordinated perioperative approach may serve as a practical reference for similarly complex, high-risk cases (29, 30).

This case provides several clinically important insights. First, although inadvertent transection of the main pulmonary artery is immediately life-threatening, survival beyond the perioperative period does not necessarily lead to acute pulmonary necrosis. The affected lung may instead be sustained by compensatory remodeling, with the development of multi-source systemic collateral circulation, including bronchial arteries and NBSAs. However, this adaptation creates a long-term risk of recurrent and potentially massive hemoptysis, especially in the presence of chronic infection. Long-term surveillance with periodic contrast-enhanced CT is therefore recommended to monitor collateral vessel evolution and related complications. Second, in patients with hemoptysis and suspected multi-source NBSA supply, comprehensive preprocedural vascular mapping is essential. Contrast-enhanced CT angiography should be performed before BAE to identify all potential feeding vessels, as incomplete embolization of NBSAs is a major cause of failure and recurrence. Third, when hemoptysis is refractory to BAE and the bleeding source is localized and resectable, surgical resection remains the only definitive treatment. These procedures are technically demanding and require a multidisciplinary approach, including careful preoperative planning, optimized anesthetic management, and readiness for advanced techniques such as intrapericardial vascular control in cases of distorted hilar anatomy. Several limitations should be acknowledged. As a single-case report, the proposed mechanisms—particularly the staged development of NBSA collateralization and the molecular drivers of angiogenesis—are based on the integration of existing literature with clinical, radiological, and pathological findings, rather than direct experimental validation, and should be considered as hypothesis-generating. In addition, the follow-up period of 6 months is limited. Although no recurrence of hemoptysis was observed, long-term outcomes remain uncertain, and late complications cannot be ruled out. Extended follow-up is warranted in similar cases. Finally, the optimal postoperative antifungal strategy, including agent selection and duration, requires further investigation.

## Conclusion

We report a unique case in which iatrogenic main pulmonary artery transection during lobectomy led to 7-year survival of the residual lung via progressive multi-source systemic collateral perfusion, with coexisting pulmonary aspergillosis, tuberculosis, and bronchiectasis acting as compounding pathological drivers. The central mechanism underlying refractory hemoptysis in this case was the development of an extensive, fragile, high-pressure collateral vascular network—comprising hypertrophied bronchial arteries and multiple NBSAs—that was further destabilized by Aspergillus-mediated vascular injury and SARS-CoV-2-triggered inflammatory activation. BAE achieved only temporary hemostasis due to the complexity of the multi-source collateral supply, and surgical resection of the non-functional right lower lobe was the only definitive curative option. This case underscores that when prolonged pulmonary artery ligation is complicated by concurrent fungal or mycobacterial infection and multi-source systemic collateralization, surgical resection—guided by a structured multidisciplinary approach—becomes the most viable treatment strategy. Clinicians should maintain a high index of suspicion for NBSA-driven hemoptysis in patients with complex post-surgical pulmonary anatomy and chronic infection and should perform comprehensive vascular mapping before any interventional procedure.

## Data Availability

The original contributions presented in the study are included in the article/supplementary material, further inquiries can be directed to the corresponding author.
